# Tumor organoids may be more suitable for clinical personalized chemotherapeutic drug screening in lung adenocarcinoma

**DOI:** 10.3389/fcell.2025.1639922

**Published:** 2025-10-02

**Authors:** Wuyang Yun, Yuyu Li, Yanlei Ge, Xiaoyun Zhang, Huifeng Liu, Wen Chen, Li Xiao

**Affiliations:** 1 Hebei North University, Zhangjiakou, China; 2 Department of Pathology, The 8th Medical Center of PLA General Hospital, Beijing, China; 3 North China University of Science and Technology Affiliated Hospital, Tangshan, China; 4 Department of Respiratory and Critical Care Medicine, The 8th Medical Center of PLA General Hospital, Beijing, China; 5 College of Pulmonary and Critical Care Medicine, Beijing Key Laboratory of Organ Transplantation and Immunology Regulatory, The 8th Medical Centre of Chinese PLA General Hospital, Beijing, China

**Keywords:** lung cancer, organoid model, chemotherapy response, resistance evolution, ClinicalPrediction, precision oncology

## Abstract

**Objective:**

The formulation of precision treatment strategies and the analysis of drug-resistance mechanisms for lung adenocarcinoma are highly dependent on *in vitro* models that can faithfully reflect tumor heterogeneity, dynamic drug responses, and tumor-stroma interactions. While existing preclinical models, such as two-dimensional (2D) adherent models and animal models, are widely used, their limitations in accurately recapitulating patient-specific microenvironments and the evolution of drug-resistant clones under chemotherapeutic pressure significantly restrict the reliability of treatment predictions.

**Methods:**

The study utilized a three-dimensional (3D) organoid model, a 2D adherent model, and an animal model constructed from the A549 cell line to dynamically monitor drug responses to chemotherapeutic treatments. We analyzed cell cycle arrest, proliferation inhibition, and the invasive regulatory features mediated by the human epidermal growth factor receptor 2(HER-2) mediated invasive regulatory features. The evolution of the resistance mutation spectrum was tracked through dynamic gene sequencing and compared with clinical resistance samples. Comparisons between two groups were performed using t-tests, while comparisons involving three or more groups were conducted using one-way analysis of variance (ANOVA).

**Results:**

In studies of four chemotherapy regimens (etoposide, paclitaxel, cisplatin, and carboplatin), organoid models showed a pharmacodynamic profile highly consistent with animal models. For drug-induced cell cycle block, the organoid model accurately replicated the animal model’s G2/M phase block. Analysis showed similar *in vitro* IC50 values for etoposide and carboplatin. Their tumor suppression rates in animal models also didn’t differ significantly (*P* > 0.05). The organoid model matched the animal model for Ki-67-mediated proliferation dynamics, HER2-mediated invasive phenotype, and early apoptosis (*P* > 0.05). Drug resistance analysis confirmed that Epidermal Growth Factor Receptor (EGFR)/HER2 mutations in the organoid model closely matched clinical resistance samples.

**Conclusion:**

The lung adenocarcinoma organoid model accurately simulates drug sensitivity and the evolution of drug resistance, providing a highly predictive *in vitro* platform for optimizing individualized chemotherapy regimens. This model is anticipated to reduce the costs associated with trial-and-error in clinical settings and to advance the development of precision tumor therapies. Keywords Lung Cancer, Organoid Model, Chemotherapy Response, Resistance Evolution, Clinical Prediction, Precision Oncology.

## 1 Introduction

Lung cancer remains a leading cause of global cancer-related morbidity and mortality, ranking among the most prevalent and deadly malignancies worldwide (Islami et al., 2024). This disease exhibits considerable heterogeneity, broadly categorized into two major subtypes: small cell lung cancer (SCLC) and non-small cell lung cancer (NSCLC). NSCLC, which constitutes the majority of cases, is further subclassified into distinct histologic variants, including adenocarcinoma, squamous cell carcinoma (SCC), large cell carcinoma, and large cell neuroendocrine carcinoma (LCNEC) (Zhu et al., 2023). Statistical projections from authoritative reports indicate a concerning epidemiological trend, with pulmonary malignancies expected to reach approximately 960,000 newly diagnosed cases in China during 2024 (National Cancer Center) (Wu et al., 2024). Notably, adenocarcinoma emerges as the predominant histopathological subtype within the non-small cell lung carcinoma cohort, accounting for over 40% of total diagnosed instances (Attal et al., 2023). Lung adenocarcinoma often presents with a few obvious symptoms diagnosed at an early stage, resulting in many patients being in its advanced stages (Shrestha et al., 2020). Although the treatment of lung adenocarcinoma has been revolutionized in recent years, with the gradual emergence of novel therapies such as targeted therapy and immunotherapy (Zhu et al., 2023). While early-stage lung cancer demonstrates improved survival, the 5-year survival rate for advanced disease stagnates at less than 5%, highlighting the unmet need for effective interventions, while early-stage lung cancer shows improved survival rates, the 5-year survival rate for advanced disease remains stagnant at less than 5%. This statistic underscores the urgent need for effective interventions, which are often hindered by genetic and somatic mutations resulting from the genetic instability of tumor cells. This instability contributes to a high degree of heterogeneity in lung cancers and leads to drug resistance in patients (Shi et al., 2020). Therefore, identifying suitable experimental models for sensitive drug screening is a promising strategy to address this challenge. Based on existing empirical treatments, conducting drug sensitivity tests for various patients and selecting the most appropriate medications are expected to enhance the efficacy and prognosis for lung cancer patients (Yuki et al., 2020).

Precision medicine for cancer emphasizes patient specificity and aims to provide personalized treatment based on the intrinsic biological information of individuals, utilizing a variety of cutting-edge medical technologies (Yuki et al., 2020). Currently, precision treatment options for lung cancer primarily depend on its histologic subtype and genetic characteristics (Caso et al., 2020), for instance, tyrosine kinase inhibitors (TKIs) are preferred for patients with mutations or fusions in genes such as the epidermal growth factor receptor (EGFR-Mutant NSCLC, 2023). In contrast, chemotherapy or combination immunotherapy is typically recommended for patients without identified mutations in common driver genes. While novel targeted therapies and immunotherapeutic agents have shown effectiveness (Salas-Benito et al., 2021), clinical resistance can develop, and the therapeutic response to the same drug can vary significantly among patients due to individual differences and tumor heterogeneity. Furthermore, most chemotherapeutic agents are cytotoxic and can cause substantial adverse effects (Kuderer et al., 2022), often leading patients to discontinue treatment due to intolerance. Anti-cancer drug screening represents a promising optimization strategy, as it aids in the development of treatment regimens designed to maximize therapeutic efficacy while minimizing toxic side effects. Chemoresistance refers to the gradual development of resistance to drugs by tumor cells following prolonged exposure to chemotherapeutic agents. This phenomenon significantly limits the efficacy of these drugs and is one of the primary causes of treatment failure (Emens et al., 2024). Chemoresistance enables tumor cells to evade the cytotoxic effects of chemotherapeutic agents through various mechanisms. The most common of these mechanisms include the overexpression of drug efflux pumps, evasion of cellular pathways, enhancement of DNA repair capabilities, and alterations in the tumor microenvironment (Marine et al., 2020). Additionally, tumor heterogeneity contributes to the development of chemoresistance, as variations in the response of different cancer cell subtypes to drugs increase the complexity and impact of treatment (Kar et al., 2024). The heterogeneity of cancers complicates the search for a universal treatment, as tumor cells can be inherently resistant to drugs or may acquire resistance during the treatment process (Ingham et al., 2025). Currently, despite the emergence of several new therapeutic strategies—such as combination chemotherapy, prophylaxis, and immunotherapy—that aim to mitigate the challenges posed by chemoresistance in lung adenocarcinoma, many obstacles remain. In the future, conducting in-depth studies on the mechanisms of chemotherapeutic potentiation against lung adenocarcinoma, particularly interventions that target these mechanisms, is one of the current focal points in the field of tumor therapy (Gu et al., 2025). By elucidating the molecular mechanisms underlying lung adenocarcinoma, researchers aim to develop more personalized and precise treatment regimens, ultimately enhancing both the treatment response rate and the survival rate of patients.

Exploratory studies on lung cancer and its drug resistance in recent years are typically categorized into vivo and *in vitro* models, including 2D adherent models, animal models, and 3D organoid models (Han et al., 2020). *In vitro* adherent cell cultures are commonly employed as models for studying histopathophysiology and drug responses; however, lung cancer cells cannot fully replicate the spatial structure of human tissues or the specificity of tumor formation and function due to limitations in their culture conditions and inherent instability (Neufeld et al., 2022; Zhao et al., 2024). Furthermore, antitumor drugs identified through traditional 2D adherent models have not consistently demonstrated effectiveness in clinical practice (Hu et al., 2021). Lung cancer cell lines are frequently engrafted into immunodeficient mice to create animal models. This reliable methodology enables the *in vivo* formation of tumor tissues and is utilized to simulate the physiological and pathological microenvironments of the human body, thereby supporting research in disease mechanisms, preclinical drug evaluation, and pharmacokinetic studies (Yi et al., 2017; Arnal-Estape et al., 2021). However, due to the inherent differences between animals and humans in physiological structure, tissue and organ function, and life maintenance, as well as the fact that mouse stromal cells can replace primitive human stromal and immune cells (Braekeveldt et al., 2016), animal models cannot accurately simulate the human pathophysiological environment. Additionally, animal experiments require longer modeling times, exhibit lower stability, and raise ethical considerations (Byrne et al., 2017). Due to the limitations of both 2D adherent models and animal models, effective new approaches are needed to develop advanced 3D models for disease modeling, drug development, and screening. Compared with traditional models, lung cancer organoids, as a new lung cancer research model, have diverse sources of cultured cells and constantly optimized and innovated culture media, while lung cancer organoids have obvious advantages in terms of construction success rate, proliferation speed, operability, and the ability to retain the heterogeneity of highly tumor-bearing patients (Xu et al., 2022), and not only that high throughput drug screening based on lung cancer organoids has been proved to be feasible and highly sensitive. The overall concordance between the drug sensitivity results and the clinical response is similar (Liu et al., 2023). This approach not only offers a research platform for an in-depth understanding of the mechanisms through which abnormal DNA methylation in early-stage lung cancer contributes to the initiation and progression of lung carcinogenesis but also holds significant potential as a vital tool for anticancer drug screening and as an alternative to animal models, thereby informing future clinical practice (Pan et al., 2021; Li et al., 2024). Based on this, the present study was conducted to explore the mechanism of drug sensitivity in lung adenocarcinoma from the perspective of the response of three lung adenocarcinoma models to lung adenocarcinoma chemotherapeutic drugs as well as the gene mutations of drug-resistant cells, and to construct a drug sensitivity model suitable for lung adenocarcinoma, which can help to improve the individualized and precise treatment for adenocarcinoma, and provide an opportunity for clinical standardization of treatment strategies. It can help to improve the individualized and precise treatment of lung adenocarcinoma and provide theoretical basis for clinical standardized treatment strategy.

## 2 Methods

### 2.1 Cell culture

The A549 cell line was purchased from Procell, China, and cultured in Ham’s F-12K (Kaighn’s) medium (Thermo, 21127022), supplemented with 10% fetal bovine serum (FBS) (Gibco, 10270106) and 1% penicillin/streptomycin solution (Gibco, 15140122). Move the cell culture dish into a cell incubator with a temperature of 37 °C and a 5% CO_2_ concentration.

### 2.2 Organoid culture

Cells were digested using trypsin (Gibco, 25200–056) containing 0.25% EDTA at 1,200 rpm for 3 min during the logarithmic growth phase. Matrigel (Corning, 354234) was then added to the cell precipitate for mixing, and 50 μL of the cell-Matrigel suspension was added to each well of a 24-well plate. The plates were placed in a 37 °C incubator to solidify for 15–30 min, with 500 μL of complete medium added per well. The medium was changed every 3 days, and the diameter of the organoids was maintained above 100 μm for passaging. The complete culture medium was composed of the following: Advanced DMEM/F12 medium (Gibco, 12634–010) supplemented with 2 mmol/L Glutamax (Gibco, 35050–061), 10 mmol/L HEPES buffer (Sigma, H4034), 100 U/mL penicillin, 5 μmol/mL Y-27632 (Selleck, S1049), 250 ng/ml R-Spondin1 (Peprotech, 120–38), 25 ng/mL FGF7 (Peprotech, 100–19), 20 ng/mL FGF10 (Peprotech, 100–26), 100 ng/mL Noggin (Peprotech, 120-10C), 1.25 mmol/L N-Acetylcysteine (Sigma, A9165), 50 μg/mL Primocin (InvivoGen, Ant-pm-1), 500 nmol/L A83-01 (Tocris, 2939), 500 nmol/L SB202190 (Selleck, S1077), and 1× B27 supplement (Gibco, 17504–44).

### 2.3 Transmission of organoids

The diameter of the organoid exceeded 100 μm. The upper layer of the medium was discarded, and a cell recovery solution (BD, 354253) was added at 4 °C for 30 min, followed by the addition of 6 mL of cold DPBS (Basal Media, B220KJ) at 4 °C for 5 min at 300 g. The retained cell sediment was then treated with TrypLE (Gibco, 12604–021) at 37 °C for 3 min. After 5 min, 6 mL of DPBS at 4 °C was added, followed by centrifugation at 300 *g* for 5 min. The supernatant was discarded, and Matrigel was added to the cell precipitate for mixing. A 50 μL aliquot of the cell-Matrigel suspension was added to each well of a 24-well plate. The plate was then placed in an incubator at 37 °C for 15–30 min, after which 500 μL of complete medium was added to each well following solidification.

### 2.4 Animal models

A549 cells in the logarithmic growth phase with a confluency of approximately 80%–90% were refreshed with fresh medium one night before collection. After trypsinization, the cells were washed twice with pre-cooled PBS (Solarbio, P1020J) to remove residual serum. The cell pellet was resuspended in PBS or serum-free medium to an appropriate concentration: for subcutaneous tumor inoculation, the cell count per injection was 1 × 10^6^ cells in a volume of 0.1–0.2 mL, corresponding to a cell suspension concentration of 1–5 × 10^7^ cells/mL. Inoculation was performed as soon as possible after cell digestion, completed within 30 min, and the cell suspension was kept on ice during the process to maintain cell viability. BALB/c-Nude mice aged 4–6 weeks and weighing approximately 16–18 g were used. The mice were housed in a specific pathogen-free (SPF) grade environment with free access to food and water. Tumor cells were implanted in the posterior-middle region of the axilla. Macroscopically visible tumors formed approximately within 2–4 weeks. A humanitarian endpoint was reached when the tumor size reached 2,000 mm^3^ or the mice exhibited obvious signs of distress. Mice were euthanized by cervical dislocation, and tumors were excised. The formula for calculating *in vivo* tumor volume was v = 0.5 × L × w^2^, while the formula for *in vitro* volume was v = 0.5 × L × w × h, where v = volume, L = length, w = width, and h = height. Researchers who measured the tumors were blinded to the treatment groups. Mice were sacrificed after receiving 2 weeks of drug treatment.

The experimental design and procedures of this study were conducted in accordance with the Regulations on the Administration of Laboratory Animals of the People’s Republic of China. Approval was obtained from the Laboratory Animal Welfare and Ethics Committee of the Academy of Military Medical Sciences, ensuring that the experimental process complied with the fundamental requirements of domestic laboratory animal management and protection. Furthermore, to guarantee the scientific rigor, transparency, and reproducibility of the animal experimental results, this study strictly adhered to the ARRIVE Guidelines, thereby effectively balancing scientific integrity and animal welfare considerations.

### 2.5 Histology and immunohistochemistry

Adherent cells and organoids were removed from the medium and washed twice with PBS. They were then digested with 0.25% trypsin-EDTA. Following this, 1 mL of 4% paraformaldehyde (PFA) was added to each well of a 24-well plate and fixed at room temperature for 30 min. After fixation, 5 mL of PBS was added, and the mixture was collected into 15 mL centrifuge tubes. The samples were centrifuged at 300 *g* for 5 min at 4 °C, and this process was repeated three times. The samples were then embedded in agarose for histological analysis, including hematoxylin and eosin (H&E) staining. Immunohistochemical staining (IHC) for CK7 (Abcam, EPR17078), Her-2 (Roche, 05999570001), and Ki-67 (Origene, 25061905) were performed according to the manufacturer’s instructions.

Mice were euthanized on days 1 and 3 following drug administration, and tumors were excised and fixed in 4% PFA for histological analysis. IHC staining for CK7, HER-2, and Ki-67 were performed according to the provided protocols.

### 2.6 TUNEL fluorescence staining

The woven paraffin sections were deparaffinized and dehydrated. The membranes were then rinsed with a Triton X-100 (Sigma, T8787) membrane-disrupting solution and subsequently washed with PBS. An appropriate amount of TDT enzyme and dUTP was mixed in a ratio of 1:50 (Sigma, 11684795910) and added to the tissue sections. The nuclei were retained with DAPI (Beyotime, C1106), sealed, and photographed under a fluorescence microscope (Nikon, Japan) to observe apoptosis. DAPI staining resulted in blue nuclei, while the nuclei of apoptotic cells were labeled green due to the positive fluorescein labeling of TUNEL. (DAPI staining shows nuclei in blue, and TUNEL fluorescein labeling indicates positive apoptotic nuclei in green).

### 2.7 Drug configuration

Etoposide (Qilu Pharmaceutical, H37023183), paclitaxel (Qilu Pharmaceutical, H20193309), cisplatin (Qilu Pharmaceutical, H20073652), and carboplatin (Qilu Pharmaceutical, H20020180) were dissolved in 0.9% saline (Sigma, 07982). Subsequently, each stock solution was subjected to ten-fold serial dilution using complete medium to prepare a total of 5 concentration gradients: 0.01, 0.10, 1.00, 10.00, and 100.00 μM. All drug solutions were freshly prepared prior to the experiment.

### 2.8 Drug sensitivity experiment

The organoids were incubated with a cell recovery solution at 4 °C for 40 min, after which digestion was terminated using cold DPBS. The mixture was then centrifuged at 4 °C for 5 min at 300 g, and the resulting pellet was separated into single cells using the organoid digest. The single cells were collected, and the cell suspension was diluted to 200 cells/μL with organoid medium containing 50% Matrigel. A volume of 20 μL of the cell mixture was seeded into each well of a 96-well plate. Once the spheres reached a diameter of 50 μm, various concentrations of the clinical drugs cisplatin and carboplatin were added. A control group with 0.9% saline was established, and dilutions with saline were prepared in five concentration gradients: 0.01, 0.10, 1.00, 10.00, and 100.00 μmol/L, with six replicate wells for each concentration. After 3 days, CCK8 reagent (Abmole, M4839) was added to assess cell Cell viability was determined by adding CCK8 reagent (Abmole, M4839) after 3 d. The data were analyzed by nonlinear curve fitting in GraphPad Prism 9, with the drug dose plotted on the horizontal axis and the cell viability values on the vertical axis.

### 2.9 Cell cycle analysis

Adherent cells in the logarithmic phase were treated with either a drug or saline for 24 h, then digested into single cells using 0.25% trypsin (Gibco, 15050–065) without EDTA. Following this, 700 µL of cold (4 °C) ethanol was added while gently vertexing to fix the cells. Organoids were similarly treated with a drug or saline for 24 h, after which they were collected and isolated into single cells. The cells were resuspended in 300 µL of cold (4 °C) PBS, and then 700 µL of cold (4 °C) 70% ethanol was added while gently vertexing to fix the cells. Mice were tumorigenic and treated with drugs or saline for 24 h; the tumors were subsequently removed and chopped into pieces the size of rice grains. Five milliliters of 5 mg/mL type II collagenase (Sigma, C2-28-100 MG) was used to digest the tissue for 40 min, after which the mixture was filtered through a 50 µm filter. Erythrocyte lysate (Solarbio, R1010) was used to lyse the cells, which were then fixed. The cells were incubated at 4 °C for at least 30 min, washed with PBS, and cell cycle analysis was performed using a DNA content quantification kit (Solarbio, CA1510) according to the manufacturer’s instructions. Data were acquired using flow cytometry (BD, United States) and analyzed with FlowJo V10 software.

### 2.10 Screening of drug-resistant cells

When the cell state was stabilized, 10 µM of the drug was added to the culture medium once the cells had reached 80% confluence. After 24 h of continuous drug exposure, the medium was replaced with drug-free medium. When cell growth reaches 80% confluence or the diameter of the organoid reaches 100 μm, passage of the cells should be performed. After passing the cells for three generations, if they remain in good condition, continue culturing with the same drug concentration. Repeat the steps and treat the cells with the drug continuously for approximately 4 weeks, during which the cells can maintain their growth in medium containing a higher concentration of the drug.

### 2.11 Drug-resistant cell gene detection

The adherent cells and organoids were digested into individual cells as described above. The analysis focused on 11 lung cancer-related mutations: EGFR, HER2, KRAS, BRAF, ALK, ROS1, RET, NTRK1, NTRK2, NTRK2 gene fusion, and MET exon 14 jumping mutations. This was conducted using the multiplexed fluorescence PCR-based Human Lung Cancer 11 Mutations Detection Kit (AmoyDx, Xiamen, China). Each sample was analyzed alongside a positive control (LET) and a negative control (purified water from NIC).

### 2.12 Statistical analyses

Results were expressed as the mean ± standard deviation (M ± SD). A t-test was employed for comparisons between two groups, while one-way analysis of variance (ANOVA) was utilized for comparisons involving three or more groups. All statistical analyses were conducted using GraphPad Prism software. Statistical significance was indicated as *P < 0.05, **P < 0.01, ***P < 0.001, and ****P < 0.0001.

## 3 Results

### 3.1 The organoid model demonstrates advantages in simulating 3d spatial structure and morpho function

**FIGURE 1:**
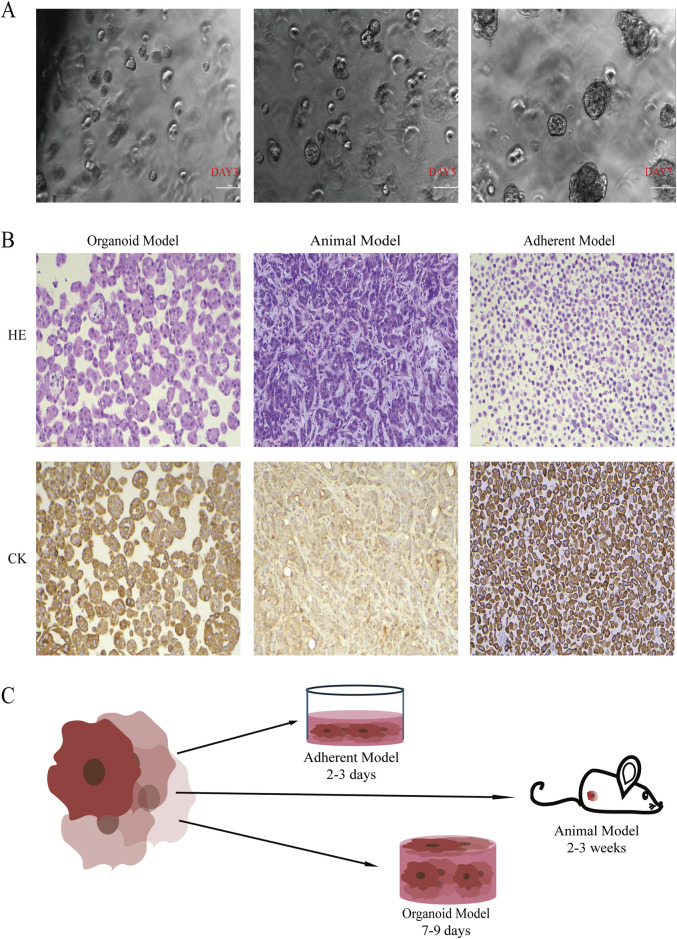
The Organoid Model Demonstrates Advantages in Simulating 3D Spatial Structure and Morpho Function. **(A)** Phase-contrast microscopy images show that the organoid diameter increased with culture time, reaching 100–200 μm by day 7 (20×, scale bar: 100 μm). **(B)** Representative HE and immunohistochemical CK7 staining for the organoid model, animal model, and adherent model. The organoid model preserved the adenomatous structure of lung adenocarcinoma and exhibited strong CK7 expression (20×, scale bar: 100 μm). **(C)** Growth cycles of models based on the A549 cell line. The organoid model completed functional construction within 9 days.

At ×20 magnification, the organoid model achieved a diameter of 100–200 µm by day 7 and maintained this size for over 3 days (Figure 1A). The organoid model exhibited adenoid structures that closely resembled those of lung adenocarcinoma, including vesicular and papillary patterns (Figure 1B). In comparison to the adherent model, tumor organoids displayed a relatively large volume, reaching up to 200 µm in diameter, and contained coarse granular chromatin. Immunohistochemical staining for CK7 confirmed the lung epithelial origin of the organoid cells (Figure 1B). In the A549 cell line-derived animal model, tumor tissues were mechanically compressed by the surrounding host tissues, resulting in a disrupted spatial arrangement of alveolar structures, which failed to fully replicate the typical alveolar morphology of primary human lung adenocarcinoma lesions (Figure 1B). HE staining of the appressed model showed only a monolayer of homogeneous cells arranged in a way that lacked the specific structural features of lung adenocarcinoma, and there was no obvious lung adenoid structure (Figure 1B). Additionally, the animal model required a prolonged growth period of 2–3 weeks. In contrast, the organoid model achieved functional construction *in vitro* within 7–9 days, demonstrating high efficiency and controllability. Although the adherent model completed growth in only 2 days, it failed to replicate the unique structure of lung adenocarcinoma (Figure 1C). In summary, interstitial fibrosis and vascular distribution in mice differ from those in human lungs, and physical compression from host tissues during tumor growth may impede alveolar structure formation. The organoid model not only recapitulates adenoid structures highly resembling lung adenocarcinoma but also offers high construction success rates and rapid proliferation.

### 3.2 Organoids mimic chemotherapy-driven cell cycle arrest in lung adenocarcinoma

**FIGURE 2:**
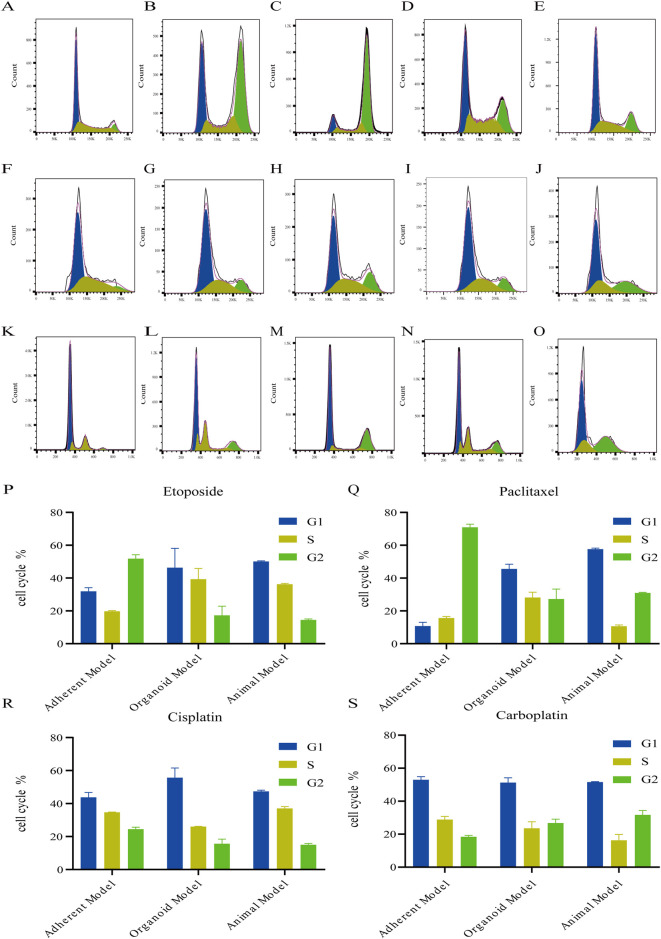
Organoids Mimic Chemotherapy-Driven Cell Cycle Arrest in Lung Adenocarcinoma. **(A–E)** Cell cycle changes in the adherent model after 24 h of treatment with 0.9% saline, etoposide (10 µM), paclitaxel (10 µM), cisplatin (10 µM), or carboplatin (10 µM),which induced obvious cell cycle disorders. Etoposide and paclitaxel predominantly caused S and G2 phase blockade. **(F–J)** The organoid model showed similar S and G2 phase cycle changes to the animal model after 24h treatment with the same chemotherapeutic agents. **(K–O)** Animal model cell cycle changes after 24h peri-tumor treatment, mainly manifesting in S and G2 phase alterations. **(P–S)** Histograms of cell cycle distributions under four chemotherapeutic agents across three models.

Table 1 as shown in Figure 2, the results demonstrated that in etoposide-treated models, the adherent cell cultures showed statistically significant differences in G1, S, and G2 phases compared to animal models, whereas organoid models exhibited no significant phase variations. Notably, significant inter-model disparities were observed between adherent and organoid systems, specifically in S and G2 phases. In paclitaxel treatment groups, adherent cultures maintained comparable S-phase characteristics with animal models, while organoids showed equivalent G2-phase profiles to *in vivo* conditions. However, adherent models displayed marked differences in both G1 and G2 phases relative to animal models. Cisplatin-treated adherent cultures significantly diverged from animal models in S and G2, phases contrasting with organoids that maintained phase consistency across G1 and G2. Similarly, carboplatin exposure induced significant S/G2 phase variations in adherent systems compared to animal models, while organoid models preserved phase stability. Collectively, these findings indicate that drug-induced cell cycle alterations in A549-derived lung adenocarcinoma organoids are more closely recapitulated *in vivo* dynamics observed in animal models compared to traditional adherent culture systems.

**TABLE 1:** Cell cycle phase distribution comparison across groups (n = 3).

Cell cycle	G1	S	G2
Adherent Model-Etoposide	31.87 (2.21)*	19.73 (0.46)**##	51.80 (2.43)**##
Organoid Model-Etoposide	46.27 (11.68)	39.27 (6.58)	17.27 (5.58)
Animal Model-Etoposide	50.07 (0.45)	36.20 (0.56)	14.47 (0.64)
Adherent Model-Paclitaxel	10.77 (2.29)▲▲##	15.63 (0.93)##	70.97 (1.89)▲▲##
Organoid Model-Paclitaxel	45.57 (2.77)▲▲	28.13 (3.25)▲▲	27.2 (5.84)
Animal Model-Paclitaxel	57.6 (0.66)	10.62 (0.84)##	30.93 (0.36)
Adherent Model-Cisplatin	43.77 (2.97)#	34.73 (0.12)□##	24.50 (1.21)□□##
Organoid Model-Cisplatin	55.73 (5.72)	26.03 (0.15)□□	15.63 (2.75)
Animal Model-Cisplatin	47.33 (0.75)	37.00 (1.18)	15.00 (0.80)
Adherent Model-Carboplatin	52.93 (1.91)	28.87 (1.93)■■	18.40 (0.90)■
Organoid Model-Carboplatin	51.20 (2.97)	23.53 (4.07)	26.77 (2.35)
Animal Model-Carboplatin	51.57 (0.25)	18.60 (7.80)	29.37 (6.66)

Compared with the etoposide-treated animal group, *P< 0.05, **P< 0.01; ▲ *P*< 0.05, ▲▲ *P*< 0.01 vs. paclitaxel group; □ *P*< 0.05, □□ *P*< 0.01 vs. cisplatin group; ■ *P*< 0.05, ■■ *P*< 0.01 vs. carboplatin group. #*P*< 0.05, ##*P*< 0.01, indicate comparison of organoid models among groups with other types of models. Results were expressed as the mean ± standard deviation (M ± SD).

### 3.3 Organoid-based drug screening to predicted chemotherapeutic drugs response *in Vivo*

**FIGURE 3:**
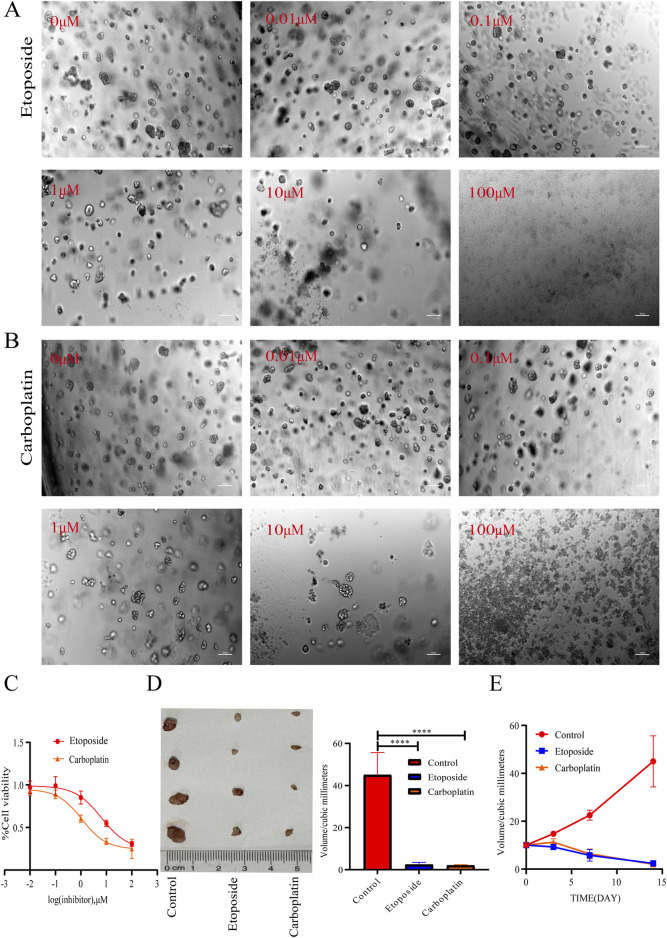
Organoid-Based drug screening to predicted chemotherapeutic drugs response *in Vivo*. **(A,B)** Phase-contrast microscopy images of organoids subjected to treatment with etoposide/carboplatin (0–100 μM) for a duration of 72 h (10×, scale bar: 100 μm). The disintegration of organoids was observed to correlate with increasing concentrations of the drugs. **(C)** Dose-response curves for etoposide and carboplatin in organoids. **(D)** Tumor xenograft volumes following a 14-day treatment regimen with saline, etoposide (10 μM), or carboplatin (10 μM) (n = 12/group). The groups treated with chemotherapeutic drugs demonstrated a significant reduction in tumor volume compared to the control group (****P < 0.0001, one-way ANOVA). **(E)** Time-dependent changes in tumor volume in mice treated with etoposide/carboplatin over periods of 3, 7, and 14 days.

As shown in Figure 3, in our previous study, we determined that the cell cycle dynamics of the etoposide and carboplatin treatment groups in the organoid model closely resemble the *in vivo* environment. Consequently, we assessed the IC50 values for etoposide and carboplatin, which were found to be 3.238 (2.813) µM and 4.296 (3.973) µM, respectively. These findings indicate that both platinum-based drugs exhibit similar sensitivity in the *in vitro* model, with no statistically significant difference observed between them. Building on this, we evaluated the *in vivo* tumor morphological response curves in mice subjected to etoposide and carboplatin treatment. The results demonstrated a gradual reduction in tumor volume over time following treatment with etoposide and carboplatin when compared to the control group. Notably, while there was no statistically significant difference in tumor volume between the etoposide and carboplatin groups at the 14-day mark, a significant difference was observed when compared to the control group. This further substantiates the notion that the 3D structure of the organoid and the tumor microenvironment may effectively mimic the drug diffusion barrier present *in vivo*, thereby providing a more accurate representation of the homogeneous cytotoxic effects of chemotherapeutic agents on tumor cells. These results reinforce the reliability of organoids in predicting chemotherapy sensitivity.

### 3.4 The organoid model accurately simulated drug-induced proliferation

**FIGURE 4:**
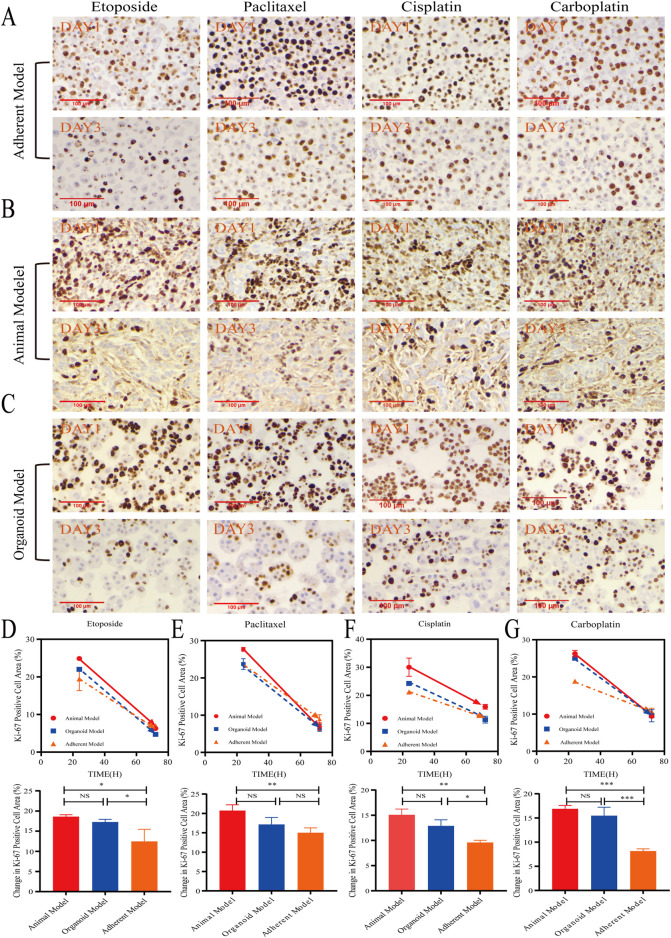
The organoid model accurately simulated drug-Induced proliferation. **(A–C)** Representative immunohistochemical images show Ki-67 staining in animal models, organoid models, and adherent models after 1 and 3 days of treatment with etoposide (10 μM), paclitaxel (10 μM), cisplatin (10 μM), and carboplatin (10 μM) (40x; scale bar: 100 µm). **(D–G)** Quantitative analysis revealed that the Ki-67+ proliferative area exhibited a time-dependent reduction across all models. Notably, organoid models demonstrated proliferation inhibition dynamics that were most closely aligned with animal models (*P* > 0.05, one-way ANOVA). In contrast, adherent models showed statistically significant differences (*P < 0.05, **P < 0.01, ***P < 0.001, one-way ANOVA).

As shown in Figure 4, Ki-67 protein is a nuclear antigen encoded by the MKI67 gene, and its elevated expression is frequently indicative of active tumor cell proliferation, increased malignancy, and poor prognosis. Results from immunohistochemical staining demonstrated that, following 1 or 3 days of treatment with four different chemotherapeutic agents, the organoid model exhibited changes in Ki-67-positive cells that were more comparable to those observed in the animal model than those in the adherent model. This study suggested that the organoid model may more effectively simulate the proliferation status of tumor cells following treatment with chemotherapeutic agents. This enhanced simulation was likely attributable to the preservation of a subpopulation of cancer stem cells (CSCs) and the proliferative signals facilitated by the three-dimensional microenvironment, which includes components such as the extracellular matrix (ECM). These factors significantly influence the sensitivity or resistance of cells to drugs. By maintaining these microenvironmental elements, the organoid model could more accurately represent the proliferation characteristics of tumor cells *in vivo*, thereby providing a closer approximation to the actual tumor environment regarding chemotherapeutic response. In contrast, the alterations in Ki-67 positivity observed in the adherent model differed from those in both the organoid and animal models. The adherent model exhibited a smaller change in proliferative activity, yet a more pronounced change in the etoposide treatment group, which may be associated with the limitations of the adherent model. Further analysis indicated that in the untreated saline control group, the area of Ki-67-positive cells increased in both the organoid and animal models, suggesting that these models sustained higher levels of tumor proliferation activity. Conversely, the area of Ki-67-positive cells decreased in the adherent model, potentially due to restrictive growth conditions by day three, which led to diminished proliferative activity (Supplementary Material).

### 3.5 HER-2 stability in organoids supported their use as a preferred platform for targeted therapies

**FIGURE 5:**
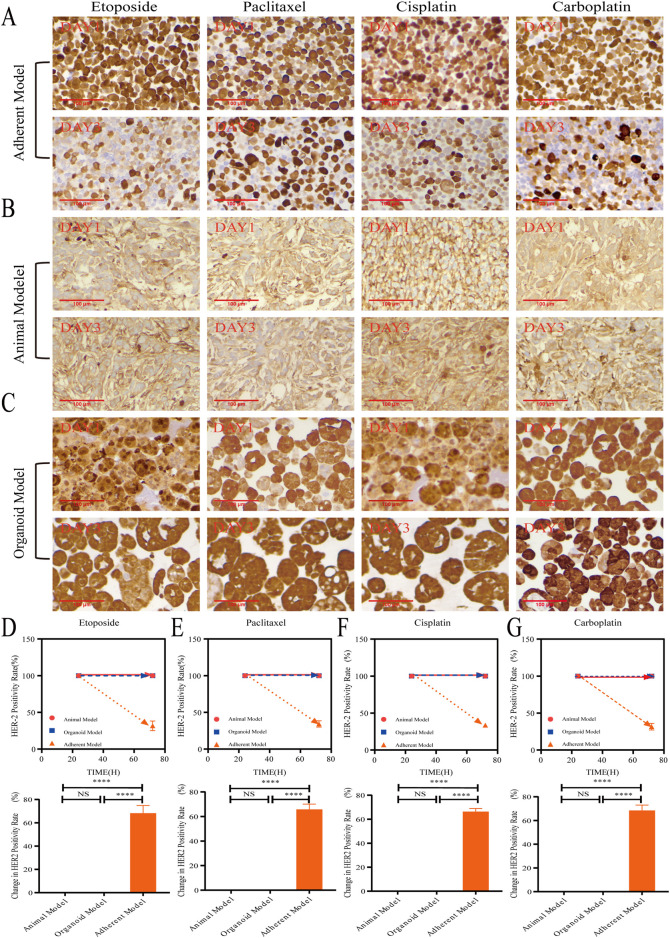
HER-2 stability in organoids supported their use as a preferred platform for targeted therapies. **(A–C)** Animal models, organoid models, and adherent models, as determined by immunohistochemical staining (40×, scale bar: 100 μm) following treatments with etoposide (10 μM), paclitaxel (10 μM), cisplatin (10 μM), and carboplatin (10 μM) over periods of 1 day and 3 days. **(D–G)** Reveal that adherent models exhibited a significant downregulation of HER-2 in response to chemotherapeutic agents (****P < 0.0001, one-way ANOVA), while organoid models maintained HER-2 membrane integrity comparable to that observed in animal models (*P* > 0.05, one-way ANOVA).

As shown in Figure 5, HER-2 is a transmembrane tyrosine kinase receptor implicated in cell proliferation, differentiation, and survival. Immunohistochemical staining results indicated that, following treatment with four chemotherapeutic agents for 1 and 3 days, the expression of HER-2 in both organoid and animal models remained unchanged despite the administration of chemotherapy. In contrast, the expression of HER-2 in the adherent model exhibited a significant decrease after 3 days of treatment when compared to the other two models. This discrepancy may be attributed to the internal hypoxia characteristic of both organoid and animal models, which activates the hypoxia-inducible factor (HIF) pathway in tumor cells, thereby influencing their metabolism, proliferation, and receptor expression, including HER-2. Conversely, the adherent model, lacking vascularization and a realistic tumor microenvironment, demonstrates a relatively homogeneous cellular growth pattern and does not experience the same hypoxic stress as the organoid or animal models. Consequently, tumor cells in the adherent model may exhibit distinct alterations in drug response and receptor expression compared to the other two models.

In conclusion, the integration of Ki-67 and HER-2 immunohistochemical staining results indicate that the response of the organoid model following chemotherapeutic treatment more closely resembled that of the animal model. This alignment allows for a more accurate representation of tumor cell proliferation and alterations in receptor expression in response to pharmacological agents. Conversely, the adherent model exhibited more pronounced variations in both Ki-67 and HER-2 indicators, which may be attributed to its distinct growth mode and microenvironmental conditions. Overall, the organoid model demonstrates significant physiological relevance in drug screening and mechanistic studies, particularly in simulating the tumor microenvironment. It offers more reliable data regarding chemotherapy responses, whereas the potential for false-positive results in the adherent model may lead to erroneous conclusions regarding drug sensitivity.

### 3.6 Organoid models accurately simulated the spatiotemporal apoptosis pattern of clinical chemotherapeutic drugs

**FIGURE 6:**
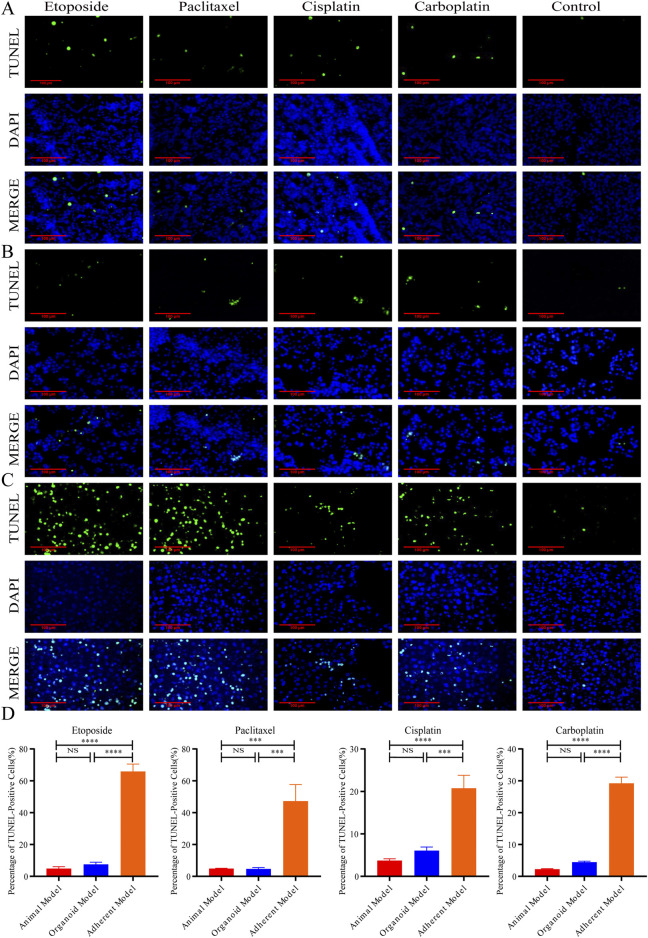
Organoid models accurately simulated the spatiotemporal apoptosis pattern of clinical chemotherapeutic drugs, **(A–C)** Representative TUNEL (green) + DAPI (blue) staining images of the animal model, organoid model, and adherent model after 24 h treatment with etoposide (10 μM), paclitaxel (10 μM), cisplatin (10 μM), carboplatin (10 μM), or 0.9% saline (40×, scale bar: 100 μm). **(D)** Apoptosis rates in the adherent model were significantly higher than in the organoid and animal models after 24 h drug treatment (***P < 0.001, and ****P < 0.0001, one-way ANOVA). No difference was observed between organoid and animal models (*P* > 0.05).

As shown in Figure 6, the results indicated that the positive rate of TUNEL staining in the adherent model was significantly higher than that observed in both the organoid model and the animal model following 24 h of treatment with etoposide, paclitaxel, cisplatin, and carboplatin, suggesting a state of hyperacute apoptosis. This finding may reflect the loss of protective tumor-stroma crosstalk in adherent cultures. In contrast, no significant difference was noted between the animal model and the organoid model, which aligns with previously reported results. Additionally, a significant increase in apoptosis was observed in peri-carcinoma cells compared to the central regions of the cancer cells under treatment with etoposide and paclitaxel, a phenomenon not replicated in the organoid model. It is plausible that in animal tumor models, the proliferative activity of cells is heightened due to the restricted blood supply in the inner regions of the tumor, the heterogeneity in drug distribution, and the typically hypoxic and nutrient-deficient environment of these inner regions. Cells located at the periphery of the tumor are generally more exposed to chemotherapeutic agents present in the bloodstream, resulting in a higher apoptosis rate among these peripheral cells. Conversely, the organoid model does not exhibit significant barriers to drug penetration, attributable to its more uniform structure and cellular arrangement. Furthermore, the absence of vascular and interstitial barriers in the organoid model, due to the lack of stromal cells and a vascular system, facilitates the uniform distribution of drugs through diffusion, leading to equivalent drug concentrations in both the inner and outer regions, and consequently, no spatial differences in apoptosis. Thus, while organoid models can more accurately predict drug efficacy and toxicity and facilitate the exploration of cellular resistance pathways, they do not adequately simulate the complexities of drug penetration, distribution, and the intricate interactions between drugs and cells (Supplementary Material).

### 3.7 Organoid modeling recapitulated the mutation profile of genes associated with clinical drug resistance in lung adenocarcinoma

Animal models exhibit limitations in their capacity to accurately replicate the heterogeneity of human tumors and the drug resistance mechanisms mediated by the tumor microenvironment. Following a 14-day period of drug administration, the tumors *in vivo* demonstrated significant reduction or complete disappearance. This rapid regression indicates that the chemotherapeutic agents employed exhibited substantial anti-tumor activity within the *in vivo* model. However, due to the inherent limitations of this model in comparison to adherent and organoid models, it is not feasible to conduct high-intensity and sustained screenings for drug-resistant cells.

**FIGURE 7:**
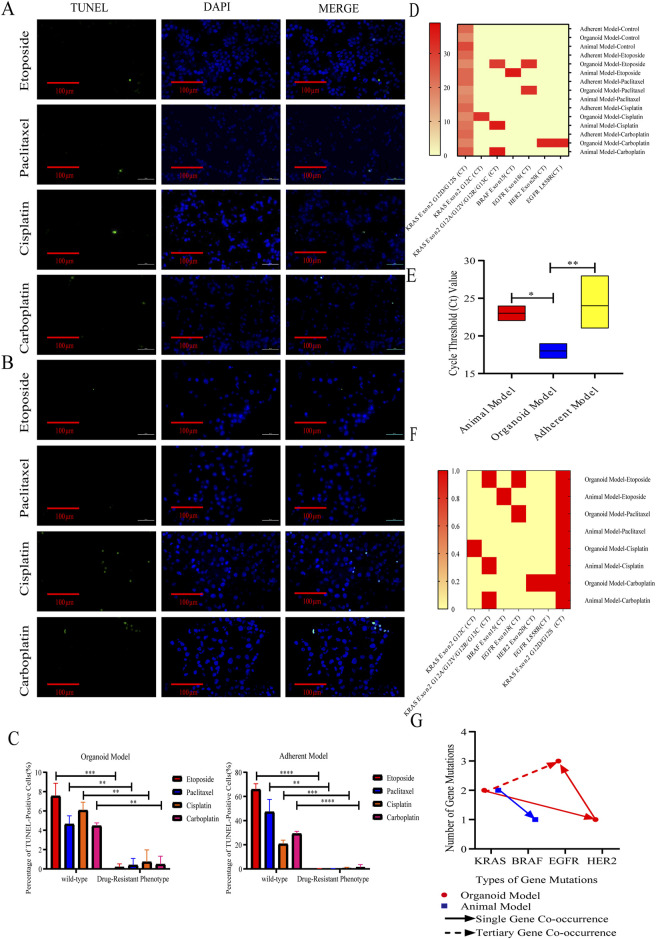
Organoid modeling recapitulated the mutation profile of genes associated with clinical drug resistance in lung adenocarcinoma. **(A,B)** Representative images (40x, scale bar: 100 µm) of TUNEL (green) and DAPI (blue) staining after administration of chemotherapeutic drugs (10 µM) to drug-resistant cells derived from organoid models and adherent models for 24 h. **(C)** Organoid model, adherent model, and apoptosis of drug-resistant types of cells in the organoid model and adherent model were significantly lower than that in the wild-type cell after screening for drug-resistant cells with 10 μM drug concentration of chemotherapeutic drugs. *P < 0.05, **P < 0.01, ***P < 0.001, T-test analysis. **(D)** Heatmap of gene mutations in drug-resistant cells in the animal model, organoid model, and adherent model, **(E)** CT values of intrinsic KRAS gene mutations in the animal model, organoid model, and adherent model, and CT values in the organoid model were significantly lower than those in the remaining two models. *P < 0.05, **P < 0.01, one-way ANOVA; **(F)** Heatmap of gene mutation co-occurrence in the animal model and organoid model, with 0 representing no mutation in the gene, and 1 representing a mutation in the gene; and **(G)** gene mutation common to the animal model and organoid model.

The results indicated that resistant cells identified through both the organoid model, and the adherent model exhibited significantly lower TUNEL positivity following chemotherapy treatment when compared to the wild-type cells (Figures 7A–C). A comparative analysis of the gene mutation characteristics across the three experimental models—namely, the animal model, the adherent model, and the organoid model—post-chemotherapy revealed that A549 cells consistently harbored mutations in the KRAS gene, specifically in Exon 2, characterized by either an aspartic acid (G12D) or serine (G12S) substitution (CT value range: 18–28). This finding suggests that KRAS mutations represent an intrinsic genetic characteristic of A549 cells and may play a pivotal role in their inherent drug resistance. Notably, no new mutations were detected in the adherent model. In the animal model, a mutation in BRAF Exon 15 was identified within the etoposide treatment group, implying that topoisomerase inhibitors may function by inducing DNA double-strand breaks and activating the bypass escape mechanism of the MAPK pathway. Additionally, novel mutations of unspecified types of KRAS were observed in the carboplatin and cisplatin treatment groups. *De novo* mutations were identified across all chemotherapy groups in the organoid model, including mutations in EGFR Exon 18, HER2 Exon 20, EGFR L858R, and KRAS G12C (Figure 7D).

The detection of six *de novo* mutations in organoids was found to be higher than that observed in animal models and adherent models. This finding suggests that organoid models are capable of capturing drug-specific genomic perturbation patterns following treatment with chemotherapeutic agents. These characteristics render organoid models superior to both adherent and animal models in elucidating mechanisms of drug resistance that are dependent on spatial heterogeneity. Furthermore, the organoid model exhibited co-mutations in EGFR and KRAS, as well as co-mutations in HER2 and EGFR, with the occurrence of EGFR and KRAS co-mutations being noted three times (Figures 7F, G). The mean CT values obtained from the organoid model were significantly lower than those from the animal and adherent models, indicating that the organoid assay is more sensitive and better suited for detecting low-frequency mutations (Figure 7E). Notably, only the organoid model demonstrated mutations in EGFR Exon 18 following paclitaxel treatment across the three models, which may be attributed to the weaker association between the drug’s target of action (the microtubule system) and DNA damage repair mechanisms. In contrast, etoposide induced novel mutations in KRAS, EGFR, and BRAF Exon 15 across all three models, reflecting a significant increase in genomic disorganization due to its interference with DNA repair processes. Additionally, the combination of carboplatin-induced EGFR L858R and HER2 mutations was observed in organoid models. These findings underscore the organoid model’s capacity to capture drug-specific genomic perturbation patterns, a phenomenon not observed in animal models. Consequently, these attributes render organoids significantly more effective than adherent and animal models in addressing spatial heterogeneity-dependent resistance mechanisms, thereby suggesting that organoids are more suitable for predicting clinical resistance patterns (Figures 7F,G).

## 4 Discussion

Lung cancer represents a significant challenge in treatment efficacy, primarily due to its complex and heterogeneous manifestations, which contribute to chemotherapy resistance. Prior research has indicated that the emergence of chemoresistance in lung adenocarcinoma is a consequence of multidimensional dynamic adaptations. These adaptations include the activation of drug efflux mechanisms, microenvironment-mediated remodeling of survival signals, and clonal evolution driven by genomic instability (Gottesman et al., 2002; Hanahan and Weinberg, 2011; McGranahan and Swanton, 2017). In the present study, we evaluated the strengths and limitations of various experimental systems in elucidating drug resistance phenotypes and mechanisms following simulated clinical drug treatment. This evaluation integrated organoid models, adherent models, and animal models, thereby providing empirical evidence for the optimization of preclinical research frameworks. While animal models demonstrate reliability in short-term assessments of drug efficacy, their inherent immunodeficiency and simplified microenvironment result in inadequate screening of drug-resistant clones (Chuprin et al., 2023). Furthermore, we observed that the rapid regression of tumors in animal models fails to capture the dynamics of clonal evolution in residual lesions post-chemotherapy, thereby constraining their utility in studies of persistent drug resistance. The adherent model is limited in its ability to replicate critical drug resistance phenotypes due to a lack of spatial heterogeneity. For instance, the rapid downregulation of HER-2 in adherent models following chemotherapy may lead to an overly optimistic evaluation of drug efficacy. This finding suggests that 2D systems are primarily suitable for the initial screening of highly sensitive drugs, whereas the intricate mechanisms of resistance necessitate the use of 3D models, such as organoids, for comprehensive analysis. Moreover, the degree of interstitial fibrosis and vascular distribution in animal models diverges from that observed in human lungs. The physical compression of host tissues during tumor growth may also disrupt the formation of alveolar structures. Consequently, the primary advantage of organoid models in investigating drug resistance mechanisms lies in their ability to preserve the key pathological features of clinical tumors. The three-dimensional architecture of organoid models accurately replicates vesicular-papillary differentiation and simulates the drug diffusion barrier and metabolic heterogeneity observed *in vivo*. Additionally, organoid models exhibit notable advantages in terms of construction success rate, proliferation speed, and overall maneuverability.

HER2, an oncogenic receptor tyrosine kinase, is frequently amplified or overactivated in various solid tumors, including breast, gastric, biliary tract, bladder, pulmonary, and gynecological cancers (Najjar et al., 2022). This molecular alteration is strongly associated with increased tumor invasiveness, accelerated malignant progression, reduced responsiveness to chemotherapy, and poor clinical outcomes characterized by early recurrence and decreased survival rates (Yan et al., 2015). In clinical practice, HER2-targeted monoclonal antibodies and tyrosine kinase inhibitors serve as the primary treatment modalities for advanced or metastatic HER2-positive breast and gastric/gastroesophageal adenocarcinomas, as well as HER2-mutant non-small cell lung cancer (NSCLC) (Cervantes et al., 2023). However, a significant proportion of patients with HER2-driven solid tumors develop either intrinsic or acquire resistance to conventional targeted therapies, leading to refractory disease trajectories with limited salvage options beyond standard treatment regimens. Our findings indicate that organoid and animal models maintain HER2 protein stability under chemotherapeutic stress, suggesting that the hypoxic core-activated HIF-1α-PI3K/AKT pathway may indirectly influence HER2 expression (Yuan et al., 2024). This observation aligns with the phenomenon whereby clinically resistant tumors evade therapeutic pressure through microenvironmental adaptation rather than through gene loss (Marine et al., 2020). Consequently, this implies that organoid models may enhance the prognosis for patients undergoing HER2-targeted therapy. Furthermore, organoid models demonstrate drug responses that are consistent with the *in vivo* environments of animal models, particularly regarding the effects of chemotherapeutic agents on cell cycle arrest, proliferation inhibition dynamics, and early spatiotemporal apoptosis. Subsequently, the dynamic evolution of the mutational profiles of resistant cells (including EGFR, HER2, and KRAS mutations) mirrors the characteristics observed in clinically resistant samples (Goto et al., 2023; Passaro et al., 2024), indicating that organoids can effectively capture drug-specific patterns of genomic perturbations. These attributes render organoids significantly superior to traditional proposals and animal models in elucidating the mechanisms of drug resistance that depend on spatial heterogeneity. Moreover, unlike the regionally heterogeneous pattern of apoptosis observed between peritumoral and intratumoral areas in animal models, apoptosis within organoid models typically demonstrates a uniform distribution. This discrepancy underscores the inherent limitations of conventional static organoid systems in accurately recapitulating the complex *in vivo* microenvironment. Notably, the absence of a functional vascular network and stromal tissue barriers in these models impedes the faithful simulation of spatial gradients in drug penetration and the heterogeneous distribution of therapeutics within tumor tissues. Such heterogeneity in drug distribution is critical, as it may modulate the activity of key apoptotic regulatory pathways—such as the glycogen synthase kinase 3β (GSK3β)/E3 ubiquitin ligase (ITCH)/cellular FLICE-like inhibitory protein (c-FLIP) axis—in a spatially dependent manner *in vivo*. For instance, within the poorly accessible tumor core, diminished drug exposure may inadequately inhibit GSK3β activity, thereby maintaining c-FLIP stability and promoting cell survival; conversely, in peripheral regions with higher drug concentrations, this pathway is more effectively suppressed, leading to increased apoptosis. Therefore, when utilizing organoid models to investigate drug resistance mechanisms mediated by this pathway, it is imperative to recognize their inability to replicate the spatiotemporal dynamics of drug distribution. This limitation may obscure the pathway’s heterogeneous regulatory functions within the authentic tumor microenvironment. Consequently, it can be concluded that the organoid model employed in this study is more appropriate for evaluating the direct cytotoxic effects of drugs and associated cell-autonomous mechanisms of drug resistance, rather than for predicting the complex *in vivo* processes related to drug delivery (Jung et al., 2019; Zheng et al., 2023).

In this study, we observed that platinum-based chemotherapeutic agents induced co-mutations in EGFR and HER2, while paclitaxel specifically resulted in variants of EGFR Exon 18, and etoposide led to mutations in both EGFR and KRAS within organoid models. These findings suggest that chemotherapy regimens may promote specific resistance pathways through epigenomic remodeling. This research offers a novel perspective on the correlation between drug treatments and mutations: by establishing a biobank of patient-derived organoids (PDOs) and employing multi-omics dynamic monitoring in response to drug perturbations, it becomes feasible to explore the complexities of tumor alterations during treatment. When PDOs are exposed to pharmacological agents, tumor cells undergo a range of molecular changes, including alterations in gene expression, variations in protein synthesis and degradation, and reconfiguration of metabolic pathways. Utilizing advanced methodologies such as high-throughput sequencing and mass spectrometry, these dynamic processes can be thoroughly monitored, facilitating the identification of early indicators and critical nodes involved in the emergence of drug resistance (Dong et al., 2024; Li et al., 2024). This approach holds promise for mapping the evolution of individualized drug resistance and guiding the design of sequential treatment protocols (Hu et al., 2021). Furthermore, the integration of organoids with microfluidic chips, along with the incorporation of immune cells and vascular endothelial components (Gerigk et al., 2021; Zhang et al., 2023) could significantly enhance the predictive capabilities of the model in simulating tumor-immune interactions and drug permeation kinetics. This integrated approach facilitates the incorporation of vascular endothelial cells into organoid co-culture systems to generate artificial microvessels and enables the simulation of dynamic drug delivery processes *via* the bloodstream through precise regulation of fluid shear stress and extracellular matrix (ECM) physical properties. Recent studies highlight that modifications in the physicochemical characteristics of the ECM are critical drivers of disease progression. A comprehensive review of research on the effects of non-enzymatic glycation (NEG) on collagen demonstrates that the accumulation of advanced glycation end products (AGEs) alters both the nanomechanical properties and biochemical signaling of collagen fibers. This methodology not only replicates the physiological barriers to drug penetration from the vasculature into tumor tissues but also permits the examination of spatial heterogeneity in drug distribution and efficacy across distinct tumor regions, such as highly proliferative and hypoxic areas. Consequently, this approach substantially enhances the model’s predictive capacity with respect to drug pharmacokinetics and pharmacodynamics (Rosenberg et al., 2023). Our findings indicate that the chemoresistance observed in A549 cells primarily depends on inherent KRAS mutations and non-genetic adaptive mechanisms. The remaining mutations identified in the three models did not meet the criteria for positivity, which may be attributed to several factors: first, conventional sequencing technologies may lack the sensitivity to reliably detect low-frequency mutations; second, the resistance phenotype may be influenced by non-mutagenic mechanisms (e.g., efflux pumps, epigenetic regulation, metabolic adaptations) rather than solely relying on DNA mutations (Leonetti et al., 2019); and lastly, low-frequency mutations may only manifest in specific microenvironments characterized by conditions such as hypoxia (Fu et al., 2021) pH variations, reductive environments, specific enzymes, reactive oxygen species (Linderman et al., 2025), and immunosuppression. The absence of a fully intact human immune system or specific substrate signaling in the three models may hinder the selection of mutant clones. Nevertheless, the organoid model demonstrated significant advantages in this study, particularly in its ability to replicate the spatial architecture of tumors *in vivo* and elucidate drug resistance mechanisms. The organoid model exhibited a superior capacity to mimic the spatial structure of tumors, with greater diversity and responsiveness in the mutation spectrum compared to both animal models and adherent growth models. Additionally, the organoid model effectively characterized the resistance mechanisms of tumor cells to chemotherapeutic agents, particularly the emergence of target mutations such as EGFR and HER2. This suggests that the organoid model may serve as a more effective platform for investigating chemotherapeutic drug resistance and accurately reproduces common mutation patterns observed in clinical settings. Moreover, the three-dimensional structure of organoids enhances their responsiveness to clinical chemotherapeutic agents, thereby more closely resembling the vivo environment.

## 5 Summary

This study has one important limitation: the use of BALB/c nude mice, which are immunodeficient. Without functional T cells, this model cannot recapitulate key immune–tumor interactions within the clinical tumor microenvironment. Consequently, the chemotherapy responses and development of drug resistance observed herein may not fully mirror the complex biological processes occurring in immunocompetent patients. For example, drug efficacy may partially rely on immune activation—such as immunogenic cell death—while resistance mechanisms could be modulated by cancer immunoediting. Nevertheless, the primary aim of this study was to directly compare, under controlled conditions, the responses to chemotherapeutic agents and associated resistance mechanisms across A549 adherent models, organoid models, and animal models. The use of an immunodeficient model was intentional to minimize immune system–derived variability, thereby enabling a clearer evaluation of inherent differences among these models in terms of drug response and genetic mutation profiles. This strategy established a well-controlled baseline that supports our central conclusion: lung adenocarcinoma organoids more faithfully replicate cell-autonomous pharmacological behaviors than traditional cell line models.

Our results strongly support the value of organoid models in lung adenocarcinoma research, particularly for resistance assessment. Organoids not only closely emulate the drug response patterns observed in animal models, but also provide practical benefits including high success rates of establishment, rapid expansion, and improved preservation of the original tumor’s spatial architecture and genetic profile. Thus, they constitute a highly promising platform for developing individualized treatment strategies. Using this model, we successfully reconstructed key molecular events during resistance evolution and uncovered preliminary dynamic patterns of tumor adaptation under drug pressure. This not only reinforces the reliability of organoids in resistance studies but also offers a novel tool for preclinical drug screening and therapy optimization.

Future efforts will focus on: 1) validating these findings in immunocompetent models (e.g., humanized mouse systems), and 2) further investigating how the tumor microenvironment influences the predictive accuracy of organoid-based drug testing to better assess clinical translatability. We will strive to develop immune-integrated organoid co-culture systems, validate results across multi-center clinical cohorts, and promote the integration of organoid technology into precision medicine for lung cancer. By addressing current model limitations and enhancing interoperability with clinical data, organoid platforms have strong potential to serve as cornerstone tools for individualized therapeutic decision-making in lung adenocarcinoma.

## Data Availability

The datasets used or analyzed in the current study are available from the corresponding authors upon reasonable request.
