# Correction: Tumor organoids may be more suitable for clinical personalized chemotherapeutic drug screening in lung adenocarcinoma

**DOI:** 10.3389/fcell.2025.1728870

**Published:** 2025-11-24

**Authors:** Wuyang Yun, Yuyu Li, Yanlei Ge, Xiaoyun Zhang, Huifeng Liu, Wen Chen, Li Xiao

**Affiliations:** 1 Hebei North University, Zhangjiakou, China; 2 Department of Pathology, The 8th Medical Center of PLA General Hospital, Beijing, China; 3 North China University of Science and Technology Affiliated Hospital, Tangshan, China; 4 Department of Respiratory and Critical Care Medicine, The 8th Medical Center of PLA General Hospital, Beijing, China; 5 College of Pulmonary and Critical Care Medicine, Beijing Key Laboratory of Organ Transplantation and Immunology Regulatory, The 8th Medical Centre of Chinese PLA General Hospital, Beijing, China

**Keywords:** lung cancer, organoid model, chemotherapy response, resistance evolution, Clinical Prediction, precision oncology

There was a mistake in Figure 1C as published.

**FIGURE 1:**
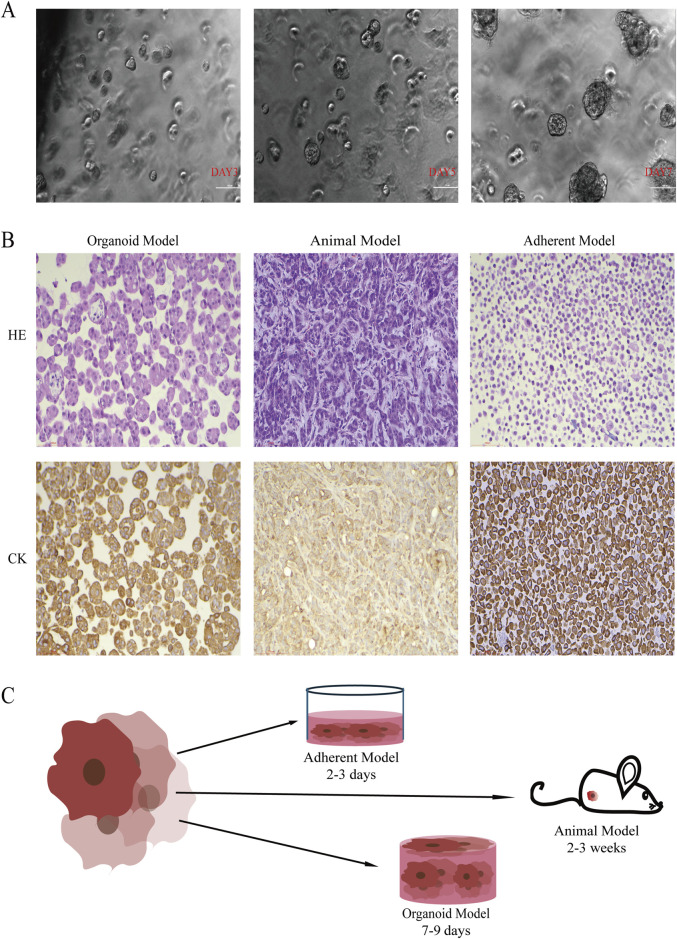
The Organoid Model Demonstrates Advantages in Simulating 3D Spatial Structure and Morpho Function. **(A)** Phase-contrast microscopy images show that the organoid diameter increased with culture time, reaching 100–200 µm by day 7 (20×, scale bar: 100 µm). **(B)** Representative HE and immunohistochemical CK7 staining for the organoid model, animal model, and adherent model. The organoid model preserved the adenomatous structure of lung adenocarcinoma and exhibited strong CK7 expression (20×, scale bar: 100 µm). **(C)** Growth cycles of models based on the A549 cell line. The organoid model completed functional construction within 9 days.

In Figure 1C the culture time for the adherent model was incorrectly stated as 2–3 weeks. The correct cultivation time for the adhesive model should be 2–3 days. The corrected Figure 1 appears below.

The original article has been updated.

