# Supplementary material for: Tumor organoids may be more suitable for clinical personalized chemotherapeutic drug screening in lung adenocarcinoma
Source: Front Cell Dev Biol. 2025 Oct 2;13:1639922. doi: 10.3389/fcell.2025.1639922 (PMC12528213; doi:10.3389/fcell.2025.1639922)
Supplement: Supplementary file 1 [file DataSheet1.docx]

**
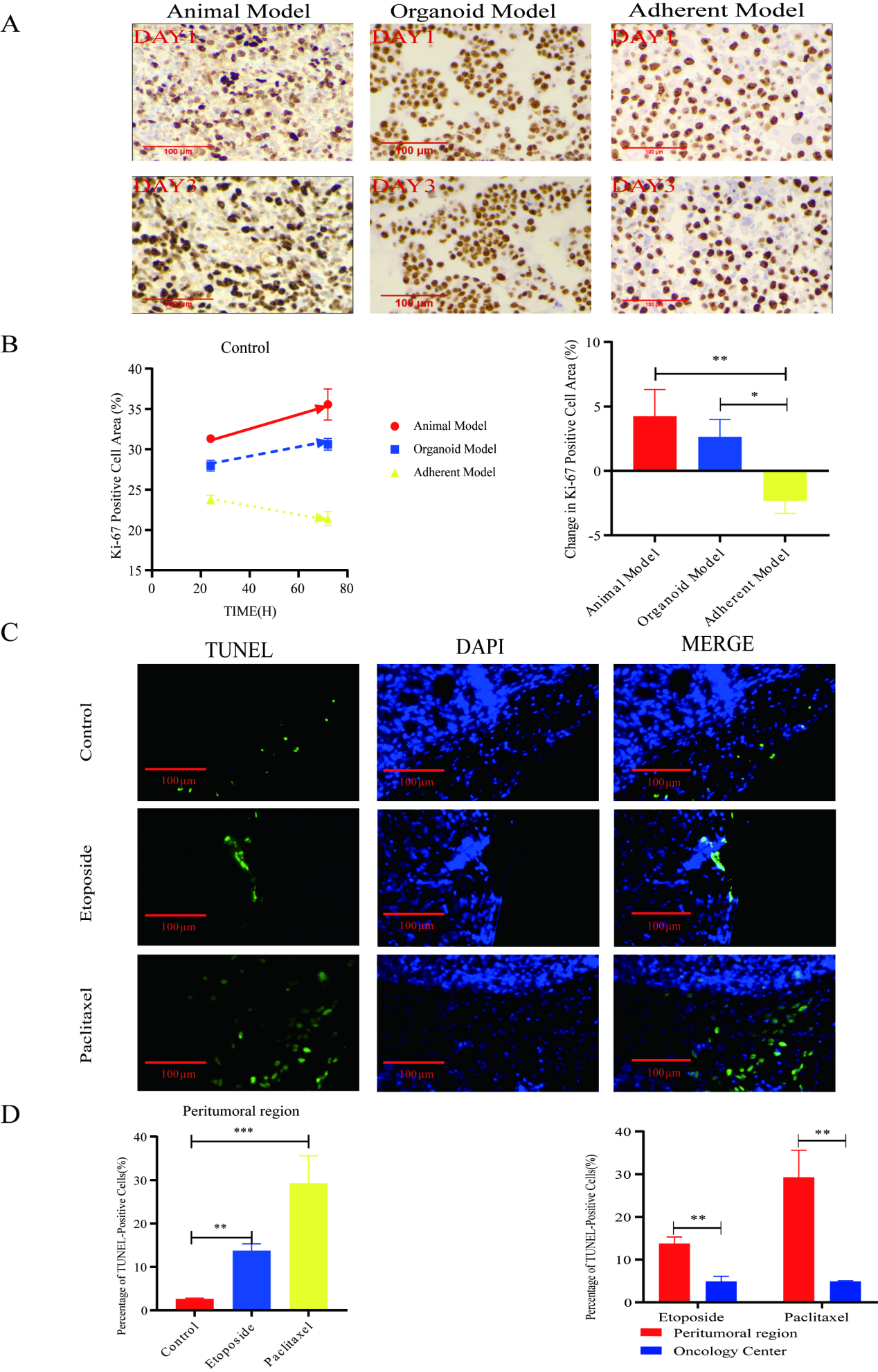
**

**Figure. S1** (A-B) Animal models, organoid models, and adherent models were treated with 0.9% saline for 1 and 3 days, and immunohistochemical images of the Ki-67 signature were obtained (20x , scale bar: 100 µm). **P*< 0.05,***P*< 0.01, one-way ANOVA. (C-D) Representative images of precancerous TUNEL (green) and DAPI (blue) results (40x , scale bar: 100 µm) after 24 hours of treatment with etoposide (10 μM) and paclitaxel (10 μM) in animal models. **P* < 0.05, ***P* < 0.01, one-way ANOVA and t-test analyses were conducted.
